# Gap-filling analysis of the *i*JO1366 *Escherichia coli* metabolic network reconstruction for discovery of metabolic functions

**DOI:** 10.1186/1752-0509-6-30

**Published:** 2012-05-01

**Authors:** Jeffrey D Orth, BernhardØ Palsson

**Affiliations:** 1 Department of Bioengineering, University of California, San Diego, La Jolla, CA, USA, 9500 Gilman Drive, Mail Code 0412, La Jolla, CA 92093-0412, USA

**Keywords:** Constraint-based modeling, Metabolic network reconstruction, *Escherichia coli*, Gap-filling, Gene annotation

## Abstract

**Background:**

The *i*JO1366 reconstruction of the metabolic network of *Escherichia coli* is one of the most complete and accurate metabolic reconstructions available for any organism. Still, because our knowledge of even well-studied model organisms such as this one is incomplete, this network reconstruction contains gaps and possible errors. There are a total of 208 blocked metabolites in *i*JO1366, representing gaps in the network.

**Results:**

A new model improvement workflow was developed to compare model based phenotypic predictions to experimental data to fill gaps and correct errors. A Keio Collection based dataset of *E. coli* gene essentiality was obtained from literature data and compared to model predictions. The SMILEY algorithm was then used to predict the most likely missing reactions in the reconstructed network, adding reactions from a KEGG based universal set of metabolic reactions. The feasibility of these putative reactions was determined by comparing updated versions of the model to the experimental dataset, and genes were predicted for the most feasible reactions.

**Conclusions:**

Numerous improvements to the *i*JO1366 metabolic reconstruction were suggested by these analyses. Experiments were performed to verify several computational predictions, including a new mechanism for growth on myo-inositol. The other predictions made in this study should be experimentally verifiable by similar means. Validating all of the predictions made here represents a substantial but important undertaking.

## Background

Constraint-based modeling is a widely used systems biology method and is particularly well suited for predicting the phenotypes of microbial organisms after gene knockouts or when grown on different substrates [1-3]. These variable conditions are simply represented as additional constraints on a model, and growth can be predicted by flux balance analysis (FBA) [4]. Because not every realistic constraint is represented in a typical metabolic model, it is quite possible for such a model to predict growth under conditions where growth does not really occur. The actual organism may not express a required gene for growth, or fluxes may be limited by kinetic or thermodynamic constraints, for example. This case is called a false positive prediction. On the other hand, false predictions of no growth can be taken as indications that the model is missing an essential reaction [5]. This prediciton is called a false negative. No current metabolic network reconstruction is entirely complete and realistic because our knowledge of the metabolism of no organism is complete. Even in very well-studied model organisms such as *Escherichia coli* there are still many genes with unknown functions [6,7]. The result of this is that there are gaps in metabolic network reconstructions. These gaps take the form of dead-end metabolites, which have either no producing or no consuming reactions [8].

Several different types of gaps can exist in reconstructed metabolic networks [8,9]. These gaps result in blocked reactions, which are unable to carry flux at steady state, and blocked metabolites, which exist only in blocked reactions and can never be produced or consumed. Root no-production gaps are metabolites that have consuming reactions but are blocked because they have no producing reactions. Metabolites that can only be produced from root no-production metabolites are also blocked, and are referred to as downstream gaps. Likewise, root no-consumption gaps are metabolites with producing reactions but no consuming reactions, and the other metabolites blocked by these gaps are called upstream gaps. The gaps in a metabolic network can also be classified as either scope gaps or knowledge gaps. Scope gaps are those that exist because the scope of most metabolic network models does not include features like macromolecular degradation or the use of charged tRNAs in protein synthesis. Knowledge gaps, on the other hand, are actually the result of our incomplete knowledge of the metabolism of any organism [10].

The comparison of model predictions to experimental data can be a useful way to fill network gaps and discover new genes and reactions. There are four possible outcomes when comparing computationally predicted to experimentally measured growth phenotypes: true positives, when the model correctly predicts growth; true negatives, when the model correctly predicts that no growth is possible; false positives, when the model predicts growth under a condition where growth was not observed; and false negatives, when the model fails to predict growth where growth was experimentally observed. Both false positive and false negative results can be useful for refining model content, but it is the false negative cases that can help fill gaps. Several methods have been developed to predict the correct gap-filling reactions based on comparisons to experimental data.

The first such method to be published was called SMILEY [5]. This is a mixed-integer linear programming algorithm that identifies the minimum number of reactions that need to be added to a metabolic model from a universal database of reactions in order to allow a minimum defined growth rate to be achieved. The SMILEY algorithm was first developed and used to predict reactions missing from the *i*JR904 *E. coli* reconstruction [11] that caused false negative model growth predictions when compared to Biolog growth data [12]. Several results were experimentally verified and new genes were characterized [5]. SMILEY was also recently used to predict gap-filling reactions in the Recon 1 human metabolic reconstruction [13,14]. The algorithms GapFind/GapFill [9] and GrowMatch [15] were later developed, and could predict missing reactions by connecting model gaps and by comparing model predictions to gene essentiality data, respectively. To date, these methods have been used to make predictions for the *E. coli* and yeast metabolic networks [15,16], but these predictions have not yet been experimentally verified. Non-constraint-based methods for reconstructing metabolic networks and filling gaps have also been developed. One example is PathoLogic, a component of the Pathway Tools software that has been used to assemble the organism specific databases of BioCyc [17]. This program fills gaps to complete metabolic pathways and even includes a hole-filling algorithm that assigns genes to gap-filling reactions [18,19]. Another recent procedure uses network expansion to determine the minimum number of reactions that need to be added to a network to make it compliant with experimental data [20]. The production of metabolites as macromolecule degradation products was considered, and genes were predicted using hidden Markov models. This strategy was applied to improve metabolic models of *E. coli*[21] and *Chlamydomonas reinhardtii*[22].

The present study builds on these methods with a new workflow that includes use of the SMILEY algorithm. SMILEY was used instead of GapFill or GrowMatch because it could be modified to make predictions for a wider range of experimental data than it was originally applied to. Specifically, it was used to make predictions using gene essentiality data and network gaps in addition to data for growth on different substrates. The *i*JO1366 metabolic network reconstruction of *E. coli* K-12, the latest and most complete genome-scale reconstruction of this organism [10], was used in this analysis. To begin, a large dataset of *E. coli* gene essentiality from the Keio Collection [23], combined from four published datasets [10,23-25], was assembled. Next, model growth predictions made using the *i*JO1366 model were compared to this dataset, and both false positive and false negative comparisons were analyzed to identify potential errors in the model and in the experimental datasets. The SMILEY algorithm was then used to predict gap-filling reactions and reactions that correct false negative model predictions. The feasibility of these reactions was then assessed by comparing augmented model predictions to the experimental dataset. Finally, genes were predicted for the most feasible putative reactions. Several sets of gene function predictions are presented, and provide plausible hypotheses for experimental validation. These predictions have the potential to improve the metabolic reconstruction and lead to new metabolic gene discoveries [8]. Knockout strain growth phenotyping experiments were performed to identify a gene involved in myo-inositol metabolism, demonstrating the types of experimental analyses that can validate these biological predictions.

## Results

### Comparison of model predictions to experimental data

**Figure 1:**
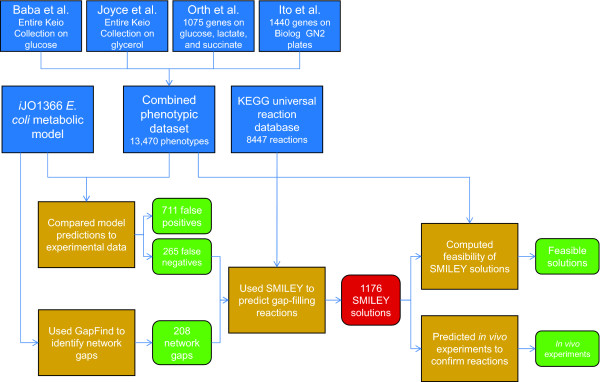
**Workflow for predicting FN-correcting and gap-filling reactions using SMILEY.** Input datasets are shown in blue, computational prediction steps are orange, and analyzed outputs are green.

By applying the developed workflow to analyze the *i*JO1366 model gaps and compare model predicted phenotypes to experimental data, new biological hypotheses were generated (Figure 1). First, the experimental datasets were assembled and combined. Each dataset consisted of a large set of *E. coli* gene knockout strains grown on different types of media. All of these gene knockout strains were from the Keio Collection of *E. coli* BW25113 single gene knockouts, allowing them to be analyzed together. The first dataset, from Baba et al. [23], contained phenotypes from the entire Keio Collection grown on glucose MOPS minimal media. This defined media contains the buffer MOPS (3-(n-morpholino)propanesulfonic acid), a potential sulfur source. The second dataset was another growth screen of the entire Keio Collection, but on glycerol M9 minimal media [25]. The third dataset was a screen of 1075 Keio Collection strains, all for genes included in the *i*AF1260 *E. coli* metabolic reconstruction [21], grown in four different media conditions [10]. The strains were grown on glucose M9 media under both aerobic and anaerobic conditions, on lactate M9 aerobically, and on succinate M9 aerobically. The fourth dataset consisted of phenotypes from 1440 Keio Collection strains grown on Biolog GN2 plates [24]. It was found that wild-type *E. coli* could grow on 38 different carbon sources on this Biolog plate, so the dataset only included these 38 substrates.

The four datasets were combined together into one large phenotypic dataset. From the screens of the entire Keio Collection on glucose and glycerol, a growth phenotype was included for each of the 1366 genes in *i*JO1366. For the screen on four conditions, phenotypes were available for 1075 of the 1366 genes. For the screen on Biolog plates, only 259 of the 1440 genes were also in *i*JO1366, so only these genes were included. Five of the 38 substrates were not included in the *i*JO1366 model or in the KEGG compound database, so these were not included since they could not be connected to the model content using the methods presented here. The phenotypes in this combined dataset were adjusted slightly from their original publications based on a more recent analysis of the Keio Collection genotypes [26]. Several new genes were classified as essential, and these were added to the essential genes on glucose and glycerol. One gene, b0103, was removed from the Biolog screen data based on this analysis. The screen of 1075 strains on four conditions was performed after the Keio Collection update, and thus already accounted for these changes. Some of the datasets contained phenotypes on the same substrates. For example, the Biolog data contained strains grown on glycerol, succinate, and lactate. In these cases, only one data point was included for each gene knockout strain grown on each substrate. If any one of the datasets included a “growth” phenotype, then the phenotype was set to “growth” in the combined dataset. Only if a strain had a “no growth” phenotype in all datasets was it classified as essential in the combined dataset. After making these adjustments, the final combined dataset contained 13,470 experimental phenotypes. There were 12,120 “growth” phenotypes and 1350 “no growth” phenotypes.

**Figure 2:**
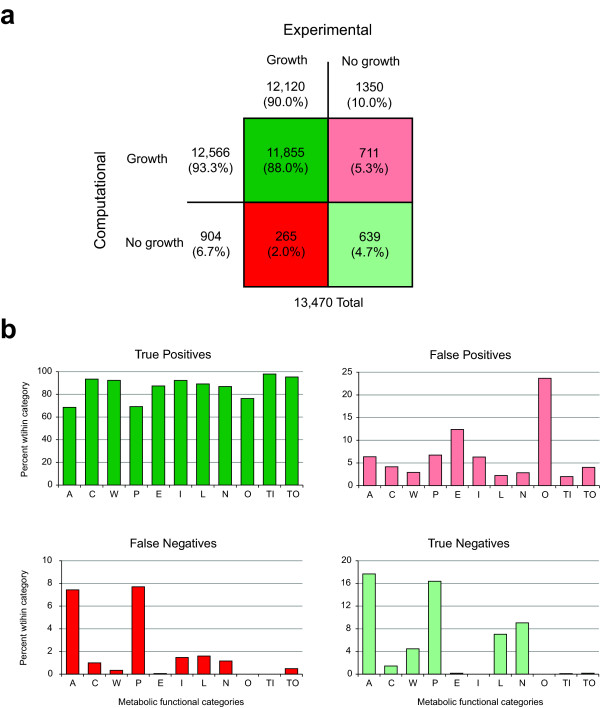
**Comparison of model predicted growth phenotypes to experimental data.** (**a**) The overall comparison, indicating numbers of true positives, true negatives, false positives, and false negatives. (**b**) The numbers of each type of prediction within 11 functional categories of metabolic reactions. The categories are: amino acid metabolism (A), carbohydrate metabolism (C), cell wall/membrane/envelope metabolism (W), cofactor and prosthetic group metabolism (P), energy production and conversion (E), inorganic ion transport and metabolism (I), lipid metabolism (L), nucleotide metabolism (N), other (O), inner membrane transport (TI), and outer membrane transport (TO).

The *i*JO1366 *E. coli* metabolic network model was then used to predict growth phenotypes for these 13,470 conditions. This model of *E. coli* K-12 MG1655 metabolism was first modified slightly to match the genotype of *E. coli* BW25113, the parent strain of the Keio Collection. FBA [4] was then used to predict growth rates using the *i*JO1366 core biomass objective with each gene knockout and on every substrate in the experimental dataset. Any growth rate above zero was classified as a computational “growth” phenotype, while a growth rate of zero was classified as “no growth”. An *in silico* dataset of 13,470 phenotypes was thus generated, and was compared to the *in vivo* dataset. Each model prediction was classified as either a true positive, true negative, false positive, or false negative. See Additional file 1 for the complete sets of computational and experimental phenotypes. A total of 11,855 true positives, 639 true negatives, 711 false positives, and 265 false negatives were identified (Figure 2 a). The Matthews Correlation Coefficient (MCC) of these predictions, a measure of the accuracy of binary classifications, was 0.5418. Overall, the *in silico* screen predicted more growth phenotypes than were found in the experimental data (93.3 % and 90.0 %, respectively). This result can largely be explained by the nature of constraint-based modeling and FBA. Because the *i*JO1366 model does not contain regulation, FBA may use any reaction in the network to produce biomass. In an *E. coli* cell, different levels of regulation may make certain enzymes unavailable under certain conditions, even if they may have allowed for growth. Other real constraints, such as kinetic or thermodynamic constraints [27], may not be accounted for in the model and also may be the cause of false positive predictions.

The genes in the *i*JO1366 model have been classified into 11 functional categories, according to the metabolic functions they serve [10]. The different categories were found to contain genes with varying levels of predictive accuracy (Figure 2 b). Genes in the “Others” category, including mainly tRNA charging genes and genes that could not be placed in the other categories, were found to lead to false positives in 23.7 % of cases. This is due to the known tRNA charging gaps in the *i*JO1366 model [10]. These tRNA charging genes are essential in vivo. There were also many false positives among the “Energy Production and Conversion” genes (12.4 %). This outcome may be partly caused by missing thermodynamic constraints, and partly by the fact that disruptions to cellular energy generation can cause *E. coli* to grow very slowly, so that these strains would have been found to be essential in the experimental screens even though they were actually slowly growing. The computational screen classified all growing strains as non-essential, even if they grew slowly. False negatives were most common among the genes in “Amino Acid Metabolism” and “Cofactor and Prosthetic Group Metabolism,” at 7.4 % and 7.7 % respectively. These false negative cases indicate the likely presence of currently unknown isozymes and alternative pathways.

### False positive model predictions

The set of false positive predictions were investigated in more detail to determine why they occurred. Every gene that had a false positive prediction on at least one substrate and had no experimental growth on any substrate was tested. It was found that there are several possible reasons for a false positive prediction to be made by the *i*JO1366 model. First, it is possible that the model may contain an error such as an unrealistic reaction (Table 1). In the model, the reaction *CBPS* (carbamoyl phosphate synthase (glutamine-hydrolyzing)) converts l-glutamine to carbamoyl phosphate, an essential precursor of l-arginine. This reaction is catalyzed by a complex of *carA* (b0032) and *carB* (b0033), which were experimentally found to be essential on glucose, glycerol, succinate, and lactate minimal media. In the model, these genes are non-essential due to an alternate reaction that produces carbamoyl phosphate, *CBMKr* (carbamate kinase), catalyzed by the products of *yahI* (b0323), *arcC* (b0521), or *yqeA* (b2874). This putative reaction is included in *i*JO1366 based only on physiological data [28], and the functions of these genes are not well characterized. It is therefore likely that the *CBMKr* reaction is unrealistic. False positives may also be caused by errors in the *i*JO1366 core biomass reaction. The gene *pdxH* (b1638) catalyzes the reactions *PDX5POi* (pyridoxine 5’-phosphate oxidase) and *PYAM5PO* (pyridoxamine 5’-phosphate oxidase), required for the synthesis of pyridoxal 5’-phosphate (vitamin B_6_). This vitamin is not included in the core biomass, so these reactions are not essential in the model. However, the essentiality of this gene on glucose and glycerol minimal media indicates that vitamin B_6_ is in fact essential to *E. coli*, and should be included in the model biomass reaction.

**Table 1:** False positive model predictions that indicate model errors

**Gene**	**Error**
*carA* (b0032)	alternate pathway (*CBMKr*) gene functions not confirmed
*carB* (b0033)	alternate pathway (*CBMKr*) gene functions not confirmed
*proB* (b0242)	alternate pathway (*NACODA*) gene function not confirmed
*proA* (b0243)	alternate pathway (*NACODA*) gene function not confirmed
*folD* (b0529)	5fthf[c] and methf[c] may be essential
*entD* (b0583)	enter[c] may be essential
*pyrD* (b0945)	alternate pathway (*DHORDfum*) is an orphan reaction
*pdxH* (b1638)	pydx5p[c] may be essential
*pgsA* (b1912)	pgp120[p] - pgp181[p] may be essential
*nrdA* (b2234)	alternate pathway (*RNDR1b* – *RNDR4b*) gene functions not confirmed
*nrdB* (b2235)	alternate pathway (*RNDR1b* – *RNDR4b*) gene functions not confirmed
*ptsI* (b2416)	alternate pathway (*GLCt2pp*) glucose transport not confirmed
*waaK* (b3623)	colipa[e] may be essential
*wzyE* (b3793)	eca4colipa[e] may be essential
*ubiE* (b3833)	reactions *AMMQLT8* and *OMBZLM* are blocked by gaps
*ubiB* (b3835)	alternate pathway (*OPXHH3*) is an orphan reaction
*ppa* (b4226)	isozymes, *ppx* (b2502) and *surE* (b2744), may be incorrect

Some false positive predictions likely occurred because a gene was incorrectly identified as essential in one of the experimental screens (Table 2). This the case with several genes involved in energy production. The cytochrome oxidase gene *cydA* (b0733) knockout strain does not exist in the Keio Collection, and is presumed to be essential. A viable knockout strain for this gene has been produced, however, along with knockouts for other cytochrome oxidases [29]. The ATP synthase genes *atpCDGAHFEB* (b3731-8) were classified as essential on minimal media, but inspection of the actual growth measurements from these experiments [10,23,25] reveals that these knockout strains did actually grow, albeit slowly.

**Table 2:** False positive model predictions that indicate incorrectly identified essential genes

**Gene**	**Reason for incorrect phenotype**
*cydA* (b0733)	knocked out successfully by Portnoy et al. [29]
*atpC* (b3731)	ATP synthase knockout causes low growth rate
*atpD* (b3732)	ATP synthase knockout causes low growth rate
*atpG* (b3733)	ATP synthase knockout causes low growth rate
*atpA* (b3734)	ATP synthase knockout causes low growth rate
*atpH* (b3735)	ATP synthase knockout causes low growth rate
*atpF* (b3736)	ATP synthase knockout causes low growth rate
*atpE* (b3737)	ATP synthase knockout causes low growth rate
*atpB* (b3738)	ATP synthase knockout causes low growth rate

Many false positive cases occurred for gene knockout strains that have known isozymes or alternative pathways (Table 3). In the *i*JO1366 model, these knockouts are overcome by using the isozyme or alternative pathway to synthesize biomass components. In vivo, these genes may be essential because isozyme genes are not expressed under the experimental conditions, or they may not be capable of catalyzing the same reaction at a sufficient rate for growth to occur. These types of false positive model predictions cannot be overcome through standard FBA using a metabolic model. A model including regulation or other additional constraints is required. Many more false positives occur when tRNA charging genes are knocked out in the model (Table 4). Since the *i*JO1366 tRNA charging reactions are blocked by scope gaps, these important reactions cannot be used in the model. Finally, several false positives cannot be explained by the model alone. For example, the gene *spoT* (b3650) is required to synthesize the signaling molecule guanosine tetraphosphate (ppGpp). Since the metabolic model does not require signaling, this gene is found to be non-essential. Experimentally, *spoT* is essential on rich media, and this is likely due to its non-metabolic function. The other false negatives that cannot be explained by the model are *ftsI* (b0084), *adk* (b0474), *mrdA* (b0635), *cydC* (b0886), *gapA* (b1779), *ligA* (b2411), *suhB* (b2533), *eno* (b2779), *fbaA* (b2925), *pgk* (b2926), *dut* (b3640), *pslB* (b4041), and *alsK* (b4084).

**Table 3:** False positive model predictions caused by isozymes or alternate pathways

**Gene**	**Isozyme or alternate pathway reactions**
*thrA* (b0002)	*metL* (b3940) or *lysC* (b4024)
*carA* (b0032)	alternate reaction: *CBMKr*
*carB* (b0033)	alternate reaction: *CBMKr*
*folA* (b0048)	*folM* (b1606)
*can* (b0126)	*cynT* (b0339)
*pyrH* (b0171)	*cmk* (b0910)
*int* (b0657)	*lpp* (b1677)
*fldA* (b0684)	*fldB* (b2895)
*fabA* (b0954)	*fabZ* (b0180)
*nrdA* (b2234)	alternate reactions: *RNDR1b*, *RNDR2b*, *RNDR3b*, *RNDR4b*
*nrdB* (b2235)	alternate reactions: *RNDR1b*, *RNDR2b*, *RNDR3b*, *RNDR4b*
*cysK* (b2414)	*cysM* (b2421)
*ptsI* (b2416)	alternate reaction: *GLCt2pp*
*cysA* (b2422)	*modA* (b0763) + *modB* (b0764) + *modC* (b0765)
*cysP* (b2425)	*modA* (b0763) + *modB* (b0764) + *modC* (b0765)
*guaB* (b2508)	alternate reaction: *XPPT*
*glyA* (b2551)	alternate reaction: *GLYCL*
*acpS* (b2563)	*acpT* (b3475)
*serA* (b2913)	alternate reaction: *GHMT2r*
*metC* (b3008)	*tnaA* (b3708) or *malY* (b1622)
*aroE* (b3281)	*ydiB* (b1692)
*ilvA* (b3772)	*tdcB* (b3117)
*metE* (b3829)	*metH* (b4019)
*ubiB* (b3835)	alternate reaction: *OPHHX3*
*glnA* (b3870)	*ycjK* (b1297)
*metL* (b3940)	*thrL* (b0002) or *malY* (b1622)
*ppa* (b4226)	*ppx* (b2502) or *surE* (b2744)
*serB* (b4388)	alternate reaction: *GHMT2r*

**Table 4:** False positive model predictions caused by tRNA charging reactions

**Gene**	**Amino Acid**
*ileS* (b0026)	L-isoleucine
*proS* (b0194)	L-proline
*cysS* (b0526)	L-cysteine
*leuS* (b0642)	L-leucine
*glnS* (b0680)	L-glutamine
*serS* (b0893)	L-serine
*asnS* (b0930)	L-asparagine
*tyrS* (b1637)	L-tyrosine
*pheT* (b1713)	L-phenylalanine
*pheS* (b1714)	L-phenylalanine
*thrS* (b1719)	L-threonine
*aspS* (b1866)	L-aspartate
*argS* (b1876)	L-arginine
*metG* (b2114)	L-methionine
*hisS* (b2514)	L-histidine
*alaS* (b2697)	L-alanine
*fmt* (b3288)	N-formyl-L-methionine
*trpS* (b3384)	L-tryptophan
*glyS* (b3559)	glycine
*glyQ* (b3560)	glycine
*valS* (b4258)	L-valine

### False negative model predictions

All genes with false negative predictions for at least one substrate and no computationally predicted growth on any substrate were investigated in more detail. If a constraint-based metabolic model fails to predict growth under a condition where growth was observed experimentally, it is an indication of missing metabolic reactions or pathways in the model. In the next section, use of the SMILEY algorithm to predict likely missing reactions is presented. There are several other possible explanations for false negative predictions. First, it is possible that the model biomass reaction being used as an objective is incorrect (Table 5). Several false negative cases occurred with knockouts of genes involved in molybdenum cofactor synthesis, including *mog* (b0009), *moaA* (b0781), *moaC* (b0783), *moaD* (b0784), *moaE* (b0785), *moeA* (b0826), *moeB* (b0827), and *mobA* (b3857). In the *i*JO1366 model, these genes are essential because they are required to produce bmocogdp[c] (bis-molybdopterin guanine dinucleotide), a component of the core biomass formulation. Because these gene knockout strains are experimentally viable on most conditions, it is likely that this cofactor is not essential for growth, and thus should not be included in the *i*JO1366 core biomass reaction.

**Table 5:** False negative model predictions caused by incorrect core biomass composition

**Gene**	**Biomass component**
*mog* (b0009)	bmocogdp[c]
*moaA* (b0781)	bmocogdp[c]
*moaC* (b0783)	bmocogdp[c]
*moaD* (b0784)	bmocogdp[c]
*moaE* (b0785)	bmocogdp[c]
*moeA* (b0826)	bmocogdp[c]
*moeB* (b0827)	bmocogdp[c]
*ubiX* (b2311)	2ohph[c]
*iscS* (b2530)	bmocogdp[c]
*cysG* (b3368)	sheme[c]
*mobA* (b3857)	bmocogdp[c]
*ubiC* (b4039)	2ohph[c]

Two false positive cases could be explained by incorrect gene-protein-reaction associations (GPRs) in *i*JO1366 (Table 6). In one, the gene *hisH* (b2023) is required for the reaction *IG3PS* (Imidazole-glycerol-3-phosphate synthase), along with *hisF* (b2025). This reaction is an essential part of the histidine synthesis pathway, and is thus essential on all minimal media for the model. In the in vivo datasets, however, *hisH* is not essential under any aerobic conditions. It is not essential because without HisH, HisF is still able to catalyze this reaction, using NH_3_ instead of glutamine as an N donor [30]. *hisH* should therefore not be an essential component of the *IG3PS* GPR. The other GPR change suggested is for *cyaY* (b3807), a gene involved in transferring iron during [Fe-S] cluster synthesis. In the model, this gene is an essential component of two reactions in both the ISC and SUF [Fe-S] cluster synthesis pathways, and is essential under all conditions. This gene is still not well characterized, and since it is experimentally non-essential, it is likely not strictly required for the reactions *I2FE2SSI2FE2SS2S2FE2SS*, and *2FE2SS2*. Other false positive cases are likely due to experimental errors (Table 7). Several genes involved in the synthesis of the cofactors biotin and thiamin were experimentally classified as non-essential. These cofactors are known to be required in small quantities [31-33], so it is likely that there was residual biotin and thiamin in the media during growth experiments. In the experimental screen on four different conditions, more thorough washing procedures were used to prevent carryover of preculture media, and these genes were classified as essential. Finally, false negatives can be caused by currently unidentified isozymes (Table 8). For cases in which false negatives could not be explained by other means, BLASTp was used to identify possible isozymes in the *E. coli* genome. One predicted isozyme has already been experimentally verified. *prpC* (b0333), which currently in the model is associated with *MCITS* (2-methylcitrate synthase), has been confirmed to also be an isozyme of *gltA* (b0720), catalyzing *CS* (citrate synthase) [34,35].

**Table 6:** **False negative model predictions that suggest changes to*i*JO1366 model GPRs**

**Gene**	**GPR correction**
*hisH* (b2023)	not essential for *IG3PS*[30]
*cyaY* (b3807)	not essential for *I2FE2SS*, *I2FE2SS2*, *S2FE2SS*, *S2FE2SS2*

**Table 7:** False negative model predictions due to misidentified experiment phenotypes or media compositions

**Gene**	**Explanation**
*mtn* (b0159)	essential according to Choi-Rhee et al. [36]
*thiI* (b0423)	possibly thiamin in media due to incomplete washing
*bioA* (b0774)	possibly biotin in media due to incomplete washing
*bioB* (b0775)	possibly biotin in media due to incomplete washing
*bioF* (b0776)	possibly biotin in media due to incomplete washing
*bioC* (b0777)	possibly biotin in media due to incomplete washing
*bioD* (b0778)	possibly biotin in media due to incomplete washing
*aroD* (b1693)	only experimental growth under one condition, possible error
*thiD* (b2103)	essential according to Orth et al. [10]
*cysD* (b2752)	only experimental growth under one condition, possible error
*argG* (b3172)	only experimental growth under one condition, possible error
*cysG* (b3368)	only experimental growth under one condition, possible error
*bioH* (b3412)	possibly biotin in media due to incomplete washing
*ilvE* (b3770)	only experimental growth under one condition, possible error
*thiH* (b3990)	essential according to Orth et al. [10]
*thiG* (b3991)	essential according to Orth et al. [10]
*thiF* (b3992)	essential according to Orth et al. [10]
*thiE* (b3993)	essential according to Orth et al. [10]
*thiC* (b3994)	essential according to Orth et al. [10]
*cysQ* (b4214)	MOPS is a possible alternate S source

**Table 8:** False negative model predictions caused by missing isozymes or alternate pathways

**Gene**	**Putative Isozyme**	**E-value**
*purK* (b0522)	*purT* (b1849)	2.00E-12
*gltA* (b0720)	*prpC* (b0333)	1.00E-41
*aspC* (b0928)	*tyrB* (b4054)	4.00E-94
*fabH* (b1091)	none identified	
*pabC* (b1096)	*ilvE* (b3770)	7.00E-8
*icd* (b1136)	*dmlA* (b1800)	3.00E-19
*aldA* (b1415)	*gabD* (b2661)	1.00E-90
	*prr* (b1444)	3.00E-80
	*feaB* (b1385)	1.00E-69
	*aldB* (b3588)	2.00E-66
	*betB* (b0312)	4.00E-65
*ubiX* (b2311)	none identified	
*luxS* (b2687)	none identified	
*thyA* (b2827)	none identified	
*zupT* (b3040)	none identified	
*folB* (b3058)	*folX* (b2303)	1.00E-4
*argG* (b3172)	none identified	
*folP* (b3177)	none identified	
*yrbG* (b3196)	none identified	
*kdsC* (b3198)	none identified	
*argD* (b3359)	*astC* (b1748)	1.00E-146
	*gabT* (b2662)	3.00E-64
	*puuE* (b1302)	3.00E-54
	*patA* (b3073)	4.00E-52
	*hemL* (b0154)	3.00E-32
*cysG* (b3368)	none identified	
*ilvE* (b3770)	*pabC* (b1096)	8.00E-8
*dapF* (b3809)	none identified	
*argC* (b3958)	none identified	
*argB* (b3959)	none identified	
*hemE* (b3997)	none identified	
*ubiC* (b4039)	none identified	
*purA* (b4177)	none identified	

### Computational prediction of gap-filling reactions

One cause of model gaps and false negative phenotypic predictions is that some realistic reactions may be missing from the *i*JO1366 model. The current version of *i*JO1366 contains 48 root no-production gaps, 63 root no-consumption gaps, 52 downstream gaps, and 69 upstream gaps. Many of these are scope gaps, caused by the limited scope of the metabolic network, and these have previously been identified [8,10]. The SMILEY algorithm was used to predict the most likely sets of reactions missing from the model. To predict false negative resolving reactions, the model was constrained to match each false negative condition, one at a time, and SMILEY was run. For gene knockout strains which lead to false negative predictions on all 34 tested substrates (or all but one or two), it is likely that the same set of missing reactions is the cause of all incorrect predictions for this strain. In these cases, SMILEY was run on the model with only glucose (both aerobic and anaerobic), glycerol, lactate, and succinate as substrates. To predict gap-filling reactions, a small lower bound was placed on the known producing or consuming reaction for each knowledge gap metabolite, and SMILEY was run. In order to actually carry a small flux through these reactions and satisfy all model constraints, a gap-filling reaction or set of reactions would need to be added. SMILEY was run on 166 false negative cases and 49 gap reactions (Additional file 2). Only model knowledge gaps [8] were targeted, not scope gaps. The algorithm was set to find up to 25 alternate solutions for each condition, and a time limit of 2 h was placed on each solution. The reactions added by SMILEY were from a universal set of reactions based on all reactions in KEGG Release 58.0 [37]. Unrealistic and incomplete reactions were removed from this set (Additional file 3).

**Figure 3:**
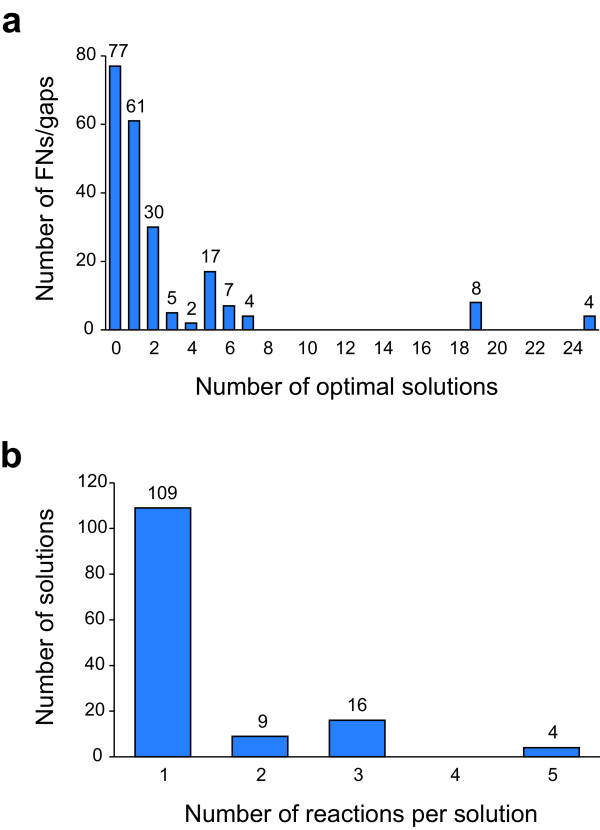
**Properties of the 198 optimal SMILEY solutions.** (**a**) Number of optimal solutions per SMILEY run. For most cases, between zero and two optimal solutions were found. (**b**) Number of reactions per optimal solution. Most optimal solutions consisted of only one reaction.

A total of 1176 optimal and suboptimal solutions were identified by SMILEY. Solutions were identified for 106 of the false negative cases and for 32 gaps. Multiple optimal solutions were found for many cases, and there were a total of 198 different optimal solutions and 983 different suboptimal solutions. Five solutions were found as both optimal and suboptimal solutions in different cases, and 385 solutions were found multiple times. Most of these were for gene knockout strains grown on multiple substrates or for genes that are required by the GPRs of the same reaction or for reactions in the same pathway. For most false negative cases and gaps, only a small number of optimal solutions were found (Figure 3 a). No solution was found for 77 cases, and only one or two solutions were found for 91 cases. In four cases, all 25 solutions were optimal. These cases were for the *iscS* (b2530) knockout strain grown on four different conditions. This gene is a part of the ISC [Fe-S] cluster generation system and in the model is essential due to its role in molybdenum cofactor synthesis. Each of these alternate solutions involves the import of this cofactor. The average number of optimal solutions found per SMILEY run was 2.56. Most optimal solutions included only one reaction, and none included more than five (Figure 3 b). The average number of reactions per optimal solution was 1.41.

As the molybdenum cofactor uptake reactions demonstrate, not all SMILEY solutions are realistic. A computational feasibility check was performed to identify the most realistic solutions. Each of the solutions was added to the *i*JO1366 model one at a time, and the augmented model was then used to predict growth phenotypes by FBA on all 13,470 conditions from the experimental dataset. The false positive and false negative predictions were identified by comparison to the experimental dataset, and the number of false negatives eliminated and new false positives created by each SMILEY solution could be counted. The most feasible solutions would be those that fixed the most false negatives while introducing few false positives. On average, each solution corrected 7.48 false negatives and created 7.07 new false positives. A total of 144 solutions (11 optimal and 133 suboptimal) were found that eliminate false negatives while producing no new false positives. GapFind was also run on the model with each solution added, to determine if any model gaps were eliminated. 74 solutions that fill at least one gap were found.

### Predictions of genes for hypothesized reactions

The most feasible SMILEY solutions out of the complete set of 1176 solutions were investigated in more detail. For the most feasible solutions, BLASTp was used to try to identify candidate genes in the *E. coli* genome (Table 9). These solutions were divided into four categories. Category I solutions were optimal solutions that eliminated at least one false negative condition while creating no new false positives. These solutions gave an average MCC of 0.5436, slightly better than the original model. Of the 11 category I solutions, five fixed false negatives by adding the deleted model reaction back in from the universal reaction list. This indicates that uncharacterized isozymes are possible for *aspC* (b0928), *pabC* (b1096), *aldA* (b1415), *argD* (b3359), and *hemE* (b3397). Another solution suggested that false negatives for Δ*aspC* strains could be corrected by adding the existing model reaction *ASP1DC* (aspartate 1-decarboxylase) in reverse. No literature evidence was found to support or refute the reversibility of this reaction. The other five solutions involve the addition of new reactions to the model. Four of these provide potential production routes for aspartate to compliment an *aspC* deletion. The other provides a new reaction to consume glycoaldehyde for Δ*aldA* strains. None of these five reactions have associated genes in the KEGG database, indicating that they are global orphan reactions. Candidate genes for these reactions could not be identified with no reference sequences available.

**Table 9:** Predicted genes for the most feasible FN-correcting SMILEY solutions

**Hypothesized changes in directionality**			
**Reaction**	**Category**	**Support**	
*ASP1DC*	I		
*ASPT*	III	reversible (Karsten and Viola [38])	
*ICL*	IV	reversible (MacKintosh and Nimmo [38])	
*AKGDH*	IV	not reversible (EcoCyc)	
*CITL*	IV		
			
**Hypothesized gap-filling reactions**			
**Reaction**	**Category**	**Putative gene**	**E-value**
R00352 (R)	IV	*sucD* (b0729)	2.00E-20
R00373 (F)	I	global orphan	
R00400 (F)	I	global orphan	
R00507 (R)	IV	*yhfW* (b3380)	0.47
R00529 (F)	IV	*cysN* (b2751) and *cysD* (b2752) *	
R00530 (F)	IV	global orphan	
R00531 (R)	IV	global orphan	
R00695 (R)	I	global orphan	
R00709 (F)	IV	*dmlA* (b1800)	6.00E-26
		*icd* (b1136)	1.00E-26
		*leuB* (b0073)	2.00E-15
R00732 (R)	III	*aroA* (b0908)	5.00E-32
		*murA* (b3189)	7.00E-8
R00733 (R)	III	*tyrA* (b2600)	2.80E-2
R01393 (R)	I	global orphan	
R01618 (R)	IV	*glgP* (b3428)	2.10
R01713 (F)	I	global orphan	
R01731 (F)	IV	*tyrB* (b4054) *	
R01785 (R)	III	*rhaD* (b3902) *	
R01902 (R)	III	*rhaB* (b3904) *	
R02200 (F)	IV	global orphan	
R04209 (R)	IV	*purC* (b2476)	7.00E-16
R05717 (R)	IV	*cysH* (b2762)	3.00E-12
R06613 (F)	II	*ybiU* (b0821)	1.6
R07164 (R)	III	*ydiJ* (b1687)	0.9
R07165 (R)	III	*ydiJ* (b1687)	0.9
R07176 (R)	IV	global orphan	
R07463 (F)	IV	*dadA* (b1189)	2.00E-18
R07613 (R)	II	*ydbL* (b0600)	7.00E-26
		*ydcR* (b1439)	6.00E-15
R08553 (R)	IV	*ysaA* (b3573)	4.00E-5

The second category of SMILEY solutions to be investigated in detail was all optimal solutions that fixed more false negatives than the number of new false positives they created. There were 70 category II solutions (not including the category I solutions, which also fall within this definition). The average MCC for these solutions was 0.5531. Most of these solutions involve the uptake of molybdenum cofactors or their precursors. As explained above, the most likely explanation for these false negatives is that the molybdenum cofactor is not strictly required for growth by *E. coli*. Several other solutions added deleted reactions back into the network, and two solutions added feasible new reactions. In one, a slightly different reaction for producing dTMP was added to compliment a *thyA* (b2827) deletion. A currently uncharacterized *E. coli* gene, *ybiU* (b0821), was identified by BLASTp as a candidate gene for this reaction, providing a testable hypothesis for the function of this gene. The other category II feasible solution added a new reaction to convert l-glutamate to α-ketoglutarate. Two candidate genes with high sequence homology to known genes from other organisms, *ydbL* (b0600) and *ydcR* (b1439), were found.

The third category to be investigated consisted of the suboptimal solutions that fixed at least one false negative while producing no new false positives. A total of 133 category III solutions were found, having an average MCC of 0.5433. Some of these solutions included unrealistic reactions, such as the oxygen consuming KEGG reaction R00357 in the reverse, oxygen producing direction. Others attempt to compensate for the loss of cofactor producing pathways by simply adding new uptake reactions for those cofactors. Still, many realistic reactions were suggested and BLASTp identified candidate genes. One solution consisted of the addition of the current model reaction *ASPT* (l-aspartase) in reverse. Experimental evidence supports the reversibility of this reaction [38], which is currently listed as irreversible in *i*JO1366. The fourth and final category of SMILEY solutions to be examined was all other optimal solutions that were not in categories I and II. There were 62 solutions in this category and they had an average MCC of 0.5304, slightly worse than the unmodified *i*JO1366 model. Most of these solutions were simply new uptake reactions for blocked essential biomass components, but 14 new realistic reactions were suggested, as well as three current model reactions running in their opposite directions. One of these new reversible reactions, *ICL* (isocitrate lyase), was confirmed in a published study [39], while another, *AKGDH* (2-Oxoglutarate dehydrogenase), is not actually reversible according to EcoCyc [40]. See Additional file 4 for all category I-IV solutions investigated.

All 72 gap-filling SMILEY solutions were also investigated (Additional file 5), and BLASTp was used to predict genes for the realistic reactions (Table 10). A total of 20 new realistic reactions were found, and candidate genes could be predicted for about half of them. The others were global orphan reactions. SMILEY also suggested that 15 existing model reactions could be made reversible to fill gaps. According to EcoCyc, some of these reactions are not reversible. However, evidence was found supporting the reversibility of two model reactions, *DKGLCNR1* (2,5-diketo-d-gluconate reductase) [41] and *DKGLCNR2y* (2,5-diketo-d-gluconate reductase (NADPH)) [42].

**Table 10:** Predicted genes for gap-filling SMILEY solutions

**Hypothesized changes in directionality**		
**Reaction**	**Support**	
*DOGULNR*	not reversible (EcoCyc)	
*DKGLCNR1*	reversible (Habrych et al. [41])	
*DKGLCNR2y*	reversible (Yum et al. [42])	
*PGLYCP*	not reversible (EcoCyc)	
*CYSSADS*		
*HMPK1*	not reversible (EcoCyc)	
*4HTHRS*		
*HETZK*	not reversible (EcoCyc)	
*NNDMBRT*	not reversible (EcoCyc)	
*ACONMT*	not reversible (EcoCyc)	
*CINNDO*	not reversible (EcoCyc)	
*MCPST*		
*GPDDAS*	not reversible (EcoCyc)	
*APCS*	not reversible (EcoCyc)	
*SARCOX*		
**Hypothesized gap-filling reactions**		
**Reaction**	**Putative gene**	**E-value**
R01742 (F)	*ydiS* (b1699)	0.003
R00893 (F)	*ygfM* (b0419)	0.58
R02133 (F)	*yhbO* (b3153)	0.069
R02721 (F)	global orphan	
R03472 (R)	global orphan	
R01297 (R)	global orphan	
R01299 (R)	global orphan	
R02252 (F)	*fadH* (b3081)	7.00E-71
	*nemA* (b1650)	1.00E-22
R00895 (R)	*aspC* (b0928)*	
R03530 (F)	*ndk* (bb2518)*	
R00012 (F)	global orphan	
R01232 (R)	*yjhG* (b4297)	0.028
	*yagF* (b0269)	0.057
R00838 (F)	*chbF* (b1734)	9.00E-42
	*melA* (b4119)	2.00E-21
R00655 (R)	global orphan	
R07300 (F)	global orphan	
R00683 (F)	global orphan	
R00367 (F)	global orphan	
R02559 (F)	global orphan	
R02560 (F)	global orphan	
R05623 (F)	*yjiN* (b4336)	0.16

### Experimental validation of predicted genes

SMILEY and other gap-filling algorithms are useful because they can use a model and existing experimental data to generate predictions. Without performing an experiment to verify these predictions, they are only hypotheses. The *i*JO1366 model was used to design simple growth phenotype experiments to confirm some of these predictions. Each of the 1176 solutions was added to the model one at a time, and growth was simulated on all combinations of a set of 115 carbon sources and 62 nitrogen sources under both aerobic and anaerobic conditions. These substrates were selected for being readily available chemicals for use in the laboratory. For every substrate combination on which growth is predicted for the model with a SMILEY solution added, but not for the unmodified *i*JO1366 model, an in vivo experiment can be performed to determine if *E. coli* can actually grow with those substrates, giving supporting experimental evidence to the predicted reactions. For most solutions, no new growth conditions were identified. All realistic solutions with testable experimental conditions are listed in Additional file 6.

**Figure 4:**
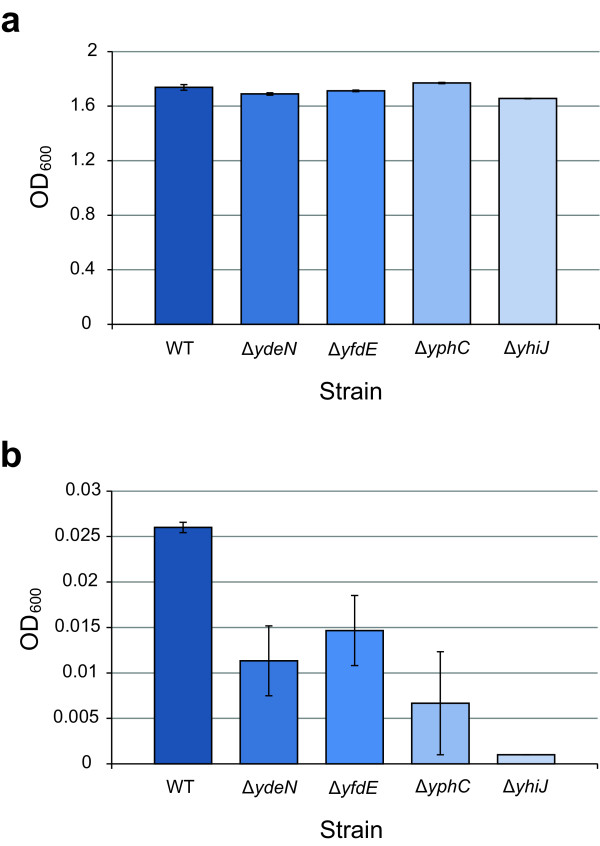
**Growth of four Keio Collection gene knockout stains to identify a possible myo-inositol:oxygen oxidoreductase.** (**a**) Growth of the four strains and WT on glucose M9 minimal media. (**b**) Growth of the four strains and WT on myo-inositol M9 minimal media. Three replicate measurements were performed.

One reaction for which a growth experiment was predicted to be possible was R01184, myo-inositol:oxygen oxidoreductase. This reaction combines myo-inositol with oxygen to form d-glucuronate and water. Myo-inositol is a root-no consumption gap in the *i*JO1366 model, and this reaction fills this gap. With this reaction included, *E. coli* is predicted to grow with myo-inositol as a substrate. An in vivo experiment was performed, and wild-type *E. coli* was inoculated into 2 g/L myo-inositol minimal media with no other carbon sources. Three replicates were performed, and after 72 h, the cultures had reached an OD_600_ of 0.017 ± 0.006. This result indicates that *E. coli* can grow very slowly with myo-inositol as its only carbon source. Next, four candidate genes were predicted for this reaction using BLASTp. These genes were *ydeN* (b1498), *yfdE* (b2371), *yphC* (b2545), and *yhiJ* (b3488). The functions of these genes are currently unknown, and they are non-essential. The Keio Collection knockout strains for these four genes were then obtained and grown in both glucose and myo-inositol minimal media. All four strains grew to a similar OD_600_ as wild-type *E. coli* on glucose, but on myo-inositol, the knockout strains did not grow as well (Figure 4). The *yhiJ* knockout strain did not grow at all, indicating this gene likely codes for a myo-inositol:oxygen oxidoreductase in *E. coli*.

## Discussion

In this study, the *i*JO1366 metabolic network model of *E. coli* was used as a discovery tool, leading to predictions for new metabolic gene functions. The most up to date model represents the current state of knowledge of *E. coli* metabolism in a structured format, and by comparing model predictions to experimental data, errors and gaps in this knowledge can be identified. A large dataset was assembled from Keio Collection gene knockout phenotypes grown on 34 different substrates. These phenotypes were compared to model predicted phenotypes, and the model false positive and false negative predictions were identified. When analyzed, the false positive predictions indicated several possible errors in the current model, including pathways thought to be catalyzed by poorly studied enzymes, and the uncertain requirements of *E. coli* biomass formation. The false negative cases also indicated several potential errors in the biomass as well as several likely experimental errors. Importantly, the false negative cases also indicated where the model is currently incomplete. The SMILEY algorithm was used to predict the most likely missing reactions, and in a novel procedure, the feasibility of these predictions was assessed through comparisons to the experimental data and through model gap analysis. Gene predictions were made for the most feasible predicted reactions, and experimental evidence was generated to support the predicted function of the gene *yhiJ*.

Through careful analysis of the false positive results, several possible model errors were identified. In some cases, the model predicted growth when pathways with poorly characterized genes were required. These genes need to be investigated in more detail. If future experimental evidence shows that they do not encode the enzymes they are currently believed to encode, then the model can be updated by removing these reactions. False negative results, on the other hand, can indicate errors in experimental design, rather than in the model. Several cases were identified in which *E. coli* could grow on minimal media despite lacking genes for the synthesis of essential cofactors. Biotin and thiamin are known to be essential, but on several substrates growth was observed for knockout strains that should not be able to produce these cofactors. One possible explanation is that there are alternate synthesis routes for these compounds, but in these cases, it is more likely that trace amounts of biotin and thiamin were present in the media during the growth experiments [23,25]. Both false positive and false negative results indicate potential errors in the *i*JO1366 core biomass reaction. False positives indicate a gene that helps produce an essential compound that is missing from the biomass reaction, while false negatives indicate a gene that produces a non-essential compound that is included. These biomass components may only be essential or non-essential under certain conditions, however, necessitating the use of condition-specific biomass reactions for specific model applications.

SMILEY was used to predict both gap-filling and false negative correcting reactions that could be added to the metabolic network. Some of these reactions were the same as existing model reactions but in the opposite direction. Literature data was searched to confirm or refute these predictions, and supporting evidence was found for several reactions. Other SMILEY solutions predicted the addition of completely new reactions to the network. All gap-filing solutions and the most feasible false negative correcting solutions were inspected manually, and for potentially realistic reactions, genes were predicted based on protein sequence homology. The most feasible predicted reactions cover a wide range of metabolic functions. Many of them corrected false negative predictions for *aspC* knockout strains, which in the model are unable to produce l-aspartate. Several others predicted new reactions involving TCA cycle intermediates. New reactions were also predicted for the metabolism of adenosine 5’-phosphosulfate, dehydroglycine, dTMP, glycoaldehyde, l-isoleucine, and 5-phosphoribosyl-5-carboxyaminoimidazole.

The new workflow presented here can theoretically be applied to any organism for which a metabolic network reconstruction is available. The only requirement is that a fairly large set of experimental phenotypes, either for growth on different substrates or for growth with gene knockouts, be available. It is also possible that this workflow could be applied using only metabolic network gaps, in the case of organisms without extensive experimental data. A similar study utilizing only gaps and not phenotypes was performed for the human metabolic network [13]. Despite the number of potentially useful predictions made, SMILEY did not find solutions for nearly half of the cases on which it was run. Part of the reason for this is that the universal set of reactions used was based on KEGG [37]. This database only contains reactions that are already known to exist in at least one organism, so completely undiscovered reactions cannot be added. Also, not every metabolite in *i*JO1366 can be connected to KEGG reactions. Of the 1133 compartment-independent metabolites in *i*JO1366, 203 do not have KEGG compound IDs. A larger set of reactions including more model metabolites would allow for additional valid SMILEY solutions to be found. Many gene predictions were made based on sequence homology, but for some reactions, no gene could be predicted because there was no reference sequence available. These are global orphan reactions [43], which have no known gene in any organism. The proliferation of global orphans (estimated to be 30-40 % of all known enzymatic functions [6]) makes gene function prediction difficult, and can account for the fact that even in a well-studied organism such as *E. coli*, there are still many uncharacterized genes.

## Conclusions

This study utilized a genome-scale metabolic network reconstruction as a tool for the analysis of high-throughput experimental data. The ultimate result of this study is that a number of valuable predictions have been made. Some of these predictions are for adjustments to the *i*JO1366 model, such as the predicted changes to the core biomass reaction and changes to the GPRs of the reactions *IG3PS*, *I2FE2SS*, *I2FE2SS2*, *S2FE2SS*, and *S2FE2SS2*. Corroborating literature evidence has been found for some of these predictions, so they should be incorporated into future updates of the reconstruction. The other predictions made through this study are for gene functions, both for isozymes and for reactions currently not known to occur in *E. coli*. These predictions provide hypotheses that can be experimentally tested. As an example, the prediction of a missing myo-inositol:oxygen oxidoreductase reaction led to the design of a simple experiment in which the previously uncharacterized gene *yhiJ* was found to be essential for growth on myo-inositol. We expect that many of the other predictions made in this study can likewise serve as hypotheses for experimental analysis.

## Methods

### Comparison of model predictions to experimental data

The experimental gene essentiality data was obtained from four publications [10,23-25], and the “essential” or “non-essential” designations assigned in the original studies were used. Several corrections to the essentiality assignments were made based on an updated analysis of the Keio Collection [26]. The newly identified essential genes were added to the lists of essential genes under all conditions, while the genes whose essentiality was identified as uncertain were not changed from their original designations.

The *i*JO1366 *E. coli* K-12 MG1655 metabolic network reconstruction was loaded into the COBRA Toolbox [44], and was adjusted to match the phenotype of *E. coli* BW25113, which is missing several metabolic genes (Δ*araBAD*, Δ*rhaBAD*, Δ*lacZ*). The associated reactions without isozymes (*ARAIRBK_L1RMPALYXIRMIRMK*, and *LACZ*) were constrained to carry zero flux. All other model reactions retained their default bounds [10]. Minimal media was simulated by setting a lower bound of −1000 (allowing unlimited uptake) on the exchange reactions for Ca^2+^, Cl^-^, CO_2_, Co^2+^, Cu^2+^, Fe^2+^, Fe^3+^, H^+^, H_2_O, K^+^, Mg^2+^, Mn^2+^, MoO_4_^2-^, Na^+^, Ni^2+^, NH_4_^-^, O_2_, HPO_4_^2-^, SeO_4_^2-^, SeO_3_^2-^, SO_4_^2-^, WO_4_^2-^, and Zn^2+^. A lower bound of −0.01 was placed on the cob(I)alamin exchange reaction. Each knockout strain was modeled by using the deleteModelGenes function to constrain the correct reactions to zero. Model growth phenotypes were determined using FBA with the core biomass reaction as the objective, one at a time on each condition. Strains with growth rates above zero were classified as non-essential, while strains with growth rates of zero were classified as essential. The Tomlab (Tomlab Optimization Inc., Seattle, WA) linear programming solver was used to perform FBA.

### Computational prediction of gap-filling reactions

The COBRA Toolbox 2.0 implementation of the SMILEY algorithm (growthExpMatch) was used to predict sets of gap-filling reactions for each false negative model comparison. The universal database of reactions was obtained from KEGG Release 58.0 [37]. All reactions in this set listed as “incomplete reaction” were blacklisted, or excluded from possible SMILEY solutions. Any reaction with the same compound appearing as both a substrate and a product was also blacklisted, along with several reactions identified in initial tests (R00090, R00113, and R00274) as forming unrealistic energy generating reaction loops with existing *i*JO1366 model reactions. The minimum growth threshold required by the SMILEY algorithm was 0.05 h^-1^. Up to 25 alternate solutions were allowed, with a single solution time limit of 2 h.

When SMILEY was run on gaps instead of false negative cases, each producing or consuming reaction for each gap metabolite was identified from the *i*JO1366 model. A lower bound of 0.01 mmol/gDW/h was applied to each reaction, one at a time, and SMILEY was used to predict gap-filling reactions. For gaps that have demand reactions in the model, the demand reactions were constrained to zero before running SMILEY. The Tomlab mixed-integer linear programming solver was used.

### Computational feasibility analysis of all predictions

After predicting sets of false negative correcting and gap-filling reactions, each of these sets of solution reactions was added to the *i*JO1366 model one at a time. The growth phenotype of each of these strains on all 13,470 experimental data conditions was then predicted using FBA, with a threshold of zero for determining growth or no growth. The number of new false positives for each solution was determined from the number of conditions that were true negatives with the wild-type model but could grow when the new reactions were added. The number of corrected false negatives for each solution was the number of false negatives that became true positives when the new reactions were added. GapFind was also run on the *i*JO1366 model with each set of solution reactions added to it, one at a time. The set of network gaps was compared to the set of gaps in the original model to determine if any gaps were eliminated.

In order to determine which SMILEY solutions could be tested with simple in vivo experiments, FBA was used to test growth of the *i*JO1366 model with each solution added on a set of 115 carbon sources and 62 nitrogen sources under both aerobic and anaerobic conditions. The growth of the unmodified *i*JO1366 model was first tested on each condition using FBA. Next, the model with each solution added, one at a time, was tested on all 3896 conditions on which the unmodified model predicted no growth. Conditions on which the modified versions of the model could grow were used to design experiments.

### Experimental validation of predicted genes

Candidate genes for SMILEY predicted reaction sets were predicted using bi-directional protein BLAST (BLASTp) between a gene from another organism in KEGG and the *E. coli* K-12 MG1655 genome. Protein sequences from organisms that are phylogenically close to *E. coli* were used when possible. The gene with the highest BLAST expectation value (E-value) found was reported. When multiple genes were found with E-values below 10^-13^, all were reported as candidate genes.

To test the growth of *E. coli* with myo-inositol as a carbon and energy source, 2 g/L myo-inositol M9 media was made and filter sterilized. This media contained M9 salts (6.8 g/L sodium phosphate dibasic, 3.0 g/L potassium phosphate monobasic, 0.5 g/L sodium chloride, 0.24 g/L magnesium sulfate, 0.011 g/L calcium chloride), trace elements (0.1 g/L iron (III) chloride, 0.02 g/L zinc sulfate, 0.004 g/L copper chloride, 0.01 g/L manganese sulfate, 0.006 g/L cobalt chloride, 0.006 g/L disodium EDTA), and Wolfe’s Vitamin Solution. Wild-type *E. coli* along with four strains from the Keio Collection with *yphC* (JW5842), *yfdE* (JW2368), *ydeN* (JW5243), and *yhiJ* (JW3455) gene knockouts (supplied by Open Biosystems) were grown overnight in LB. The next day, 15 mL of each culture was centrifuged at 4000 rpm for 8 min, the supernatant was discarded, and the culture was resuspended in an M9 salt solution with no carbon or nitrogen sources. The culture was centrifuged and resuspended in new M9 four more times to completely wash out all LB. Next, 1 μL of washed *E. coli* cultures were then used to inoculate 10 mL aerobic myo-inositol M9 cultures, which were grown at 37°C. The optical density at 600 nm was measured at several points during growth.

## Competing interests

The authors declare that they have no competing interests.

## Authors’ contributions

JDO and BOP designed the study. JDO performed all computational predictions, analyzed the results, performed the experimental analysis, and wrote the manuscript. JDO and BOP revised the manuscript. Both authors approved the content of the final manuscript.

## Supplementary Material

Additional file 1 Experimental and computational growth phenotypes for all 13,470 conditions. Click here for file

Additional file 2 All false negative conditions and gaps on which SMILEY was run. Click here for file

Additional file 3 The KEGG based universal database of reactions and list of excluded reactions. Click here for file

Additional file 4 All Category I-IV SMILEY solutions. Click here for file

Additional file 5 All gap-filling SMILEY solutions. Click here for file

Additional file 6 Media conditions that can indicate the presence of predicted reactions. Click here for file
